# Multivariate Optimization of the FLC-dc-APGD-Based Reaction-Discharge System for Continuous Production of a Plasma-Activated Liquid of Defined Physicochemical and Anti-Phytopathogenic Properties

**DOI:** 10.3390/ijms22094813

**Published:** 2021-05-01

**Authors:** Anna Dzimitrowicz, Piotr Jamroz, Pawel Pohl, Weronika Babinska, Dominik Terefinko, Wojciech Sledz, Agata Motyka-Pomagruk

**Affiliations:** 1 Department of Analytical Chemistry and Chemical Metallurgy, Wroclaw University of Science and Technology, 27 Wybrzeze St. Wyspianskiego, 50-370 Wroclaw, Poland; piotr.jamroz@pwr.edu.pl (P.J.); pawel.pohl@pwr.edu.pl (P.P.); dominik.terefinko@pwr.edu.pl (D.T.); 2 Laboratory of Plant Protection and Biotechnology, Intercollegiate Faculty of Biotechnology University of Gdansk and Medical University of Gdansk, University of Gdansk, 58 Abrahama, 80-307 Gdansk, Poland; weronika.babinska@phdstud.ug.edu.pl (W.B.); wojciech.sledz@biotech.ug.edu.pl (W.S.)

**Keywords:** non-thermal atmospheric pressure plasma, reactive oxygen and nitrogen species, *Pectobacteriaceae*, *Dickeya* spp., *Pectobacterium* spp., antibacterial, plant protection, agriculture

## Abstract

To the present day, no efficient plant protection method against economically important bacterial phytopathogens from the *Pectobacteriaceae* family has been implemented into agricultural practice. In this view, we have performed a multivariate optimization of the operating parameters of the reaction-discharge system, employing direct current atmospheric pressure glow discharge, generated in contact with a flowing liquid cathode (FLC-dc-APGD), for the production of a plasma-activated liquid (PAL) of defined physicochemical and anti-phytopathogenic properties. As a result, the effect of the operating parameters on the conductivity of PAL acquired under these conditions was assessed. The revealed optimal operating conditions, under which the PAL of the highest conductivity was obtained, were as follows: flow rate of the solution equaled 2.0 mL min^−1^, the discharge current was 30 mA, and the inorganic salt concentration (ammonium nitrate, NH_4_NO_3_) in the solution turned out to be 0.50% (*m*/*w*). The developed PAL exhibited bacteriostatic and bactericidal properties toward *Dickeya solani* IFB0099 and *Pectobacterium atrosepticum* IFB5103 strains, with minimal inhibitory and minimal bactericidal concentrations equaling 25%. After 24 h exposure to 25% PAL, 100% (1−2 × 10^6^) of *D. solani* and *P. atrosepticum* cells lost viability. We attributed the antibacterial properties of PAL to the presence of deeply penetrating, reactive oxygen and nitrogen species (RONS), which were, in this case, OH, O, O_3_, H_2_O_2_, HO_2_, NH, N_2_, N_2_^+^, NO_2_^−^, NO_3_^−^, and NH_4_^+^. Putatively, the generated low-cost, eco-friendly, easy-to-store, and transport PAL, exhibiting the required antibacterial and physicochemical properties, may find numerous applications in the plant protection sector.

## 1. Introduction

Plant diseases remain a constant threat to agriculture, forestry, and food processing [1]. Among bacterial phytopathogens of the highest economic importance, *Pectobacterium* and *Dickeya* spp. from the *Pectobacteriaceae* family [2] are often listed [3,4,5,6,7]. These microorganisms affect various crops, vegetables, and ornamental plants with the symptoms of soft rot and/or blackleg [8,9]. The ubiquitous presence [10,11] of soft rot *Pectobacteriaceae* (SRP) is responsible for significant economic losses, especially in the European potato production sector that suffers each year from a 46 M euro damage [12].

It is worth considering that no efficient control measures against *Pectobacterium* and *Dickeya* spp. have been introduced to the market yet [13] even though a vast number of potential SRP control procedures involving physical (hot water, steam, hot dry air, and UV light), chemical (antibiotics, hydrogen peroxide, essential oils, nanoparticles, antimicrobial peptides, organic, and inorganic compounds) in addition to biological (antagonistic bacteria, fungi, and lytic phages) treatments have been studied [13,14]. Unfortunately, since none of the above-mentioned approaches fulfilled all the requirements of effectiveness, high-throughput, low cost, and no damage to the plant host or environment, therefore, they have not been implemented into practice [13,14].

In search for novel methods to combat SRP, we focused our attention on non-thermal atmospheric pressure plasmas (NTAPs) [15]. NTAPs could be applied against microorganisms either directly or indirectly [16]. Direct action of NTAP requires transportation of the usually non-portable plasma source and is believed to be expensive in exploitation. Besides, such exposure might lower the quality of agricultural goods, i.e., lead to changes in color, surface topography, or diminishment in the contents of bioactive compounds [17]. Hence, we developed an indirect NTAP-based SRP eradication method based on the usage of an easy-to-store and eco-friendly plasma-activated liquid (PAL).

The contact of NTAP with an aqueous solution triggers differences in its pH, redox potential, and conductivity. In addition, it leads to a variation in the profile and the concentration of reactive oxygen and nitrogen species (RONS) [17]. Numerous long-lived RONS, e.g., hydrogen peroxide (H_2_O_2_), nitrate (NO_3_^−^), ammonium (NH_4_^+^), and nitrite (NO_2_^−^), in addition to short-lived reactive species such as hydroxyl radicals (•OH), peroxynitrite anions (ONOO^−^), atomic oxygen (O), nitrogen oxide radicals (•NO), and superoxide anions (O_2_•^−^) of well-documented antimicrobial properties are produced in such a medium [18]. The type and the concentration of the above-listed RONS might be linked with the operating parameters of NTAPs, including plasma source, the discharge atmosphere, the current-voltage characteristics, the NTAP exposure time (in the case of stationary reaction-discharge systems), or the introduced solution flow rate (concerning continuous-flow reaction-discharge systems).

Several previous works aimed at the exploitation of RONS enclosed in PALs [19] for the inactivation of microorganisms. For instance, antibacterial action of PALs has been reported before against the model microorganism *Escherichia coli* [20,21,22], human and animal pathogens like *Staphylococcus aureus* [21,23], *Bacillus cereus* [24], *Enterococcus faecalis* [25], *Klebsiella pneumoniae* [22], *Acinetobacter baumannii* [22], *Pseudomonas aeruginosa* [22], common food spoilage agents such as *Listeria monocytogenes* [26], *Leuconostoc mesenteroides* [27], *Hafnia alvei* [27], and Salmonella Typhimurium [26] treated as planktonic cells [20,21,24,26,27] in the form of complex biofilm 3D structures [21,22,25,26,27,28] or as food products’ surface contaminants [23,29,30,31,32,33]. However, little attention has been attributed to evaluating the potency of PALs for the eradication of plant pathogens instead of the food spoilage-agents [34]. So far, plasma activated waters (PAWs) have been tested against *Colletotrichum gloeosporioides* [35] causing bitter rot in numerous crops, *Xanthomonas vesicatoria* responsible for the leaf spot on tomatoes [36], *Colletotrichum alienum* being a post-harvest pathogen of avocado [37], and *Penicillium italicum* triggering blue and green molds on citrus fruits [38]. Concerning SRP, to the best of our knowledge, solely a direct impact of plasma generated either by electric discharges in a gliding arc reactor in a stationary phase [39] or a direct current atmospheric pressure glow discharge obtained in contact with a flowing cathode [40] has been reported as an effective method to combat these pests.

Even though water (tap, deionized, distilled [37]) is most commonly utilized for the generation of PALs of antimicrobial properties, phosphate-buffered saline, saline, and other NTAP-treated media [41] have also been investigated. In this work, according to the suggestions of Graves et al. [42], we aimed at combining the antibacterial action of PALs with nutritious features of a plasma-activated fertilizer. Having in mind that nitrogen fertilizers are most frequently utilized for boosting the yield and quality of the crops [43], we decided to base the formulation of the herein-reported PAL on an ammonium nitrate (NH_4_NO_3_) aqueous solution.

It is worth considering that various NTAP sources, including atmospheric pressure plasma jet [44], gliding arc electric discharge [27], and transient spark discharge [45,46] have been employed so far to generate PALs of documented antimicrobial properties. Unfortunately, the majority of the already conducted studies described the production of PALs in stationary NTAP-based reaction-discharge systems, which means that solely a predefined volume of a liquid has been exposed to the plasma [27,44]. Regarding a circle working mode, as far as we are aware, only transient spark discharge-based reaction-discharge systems [45,46] or streamer corona discharge-based systems [46] have been implemented in such a way for the acquisition of PALs [45,46]. Notably, no high-throughput reaction-discharge system, employing direct current atmospheric pressure glow discharge in contact with a flowing liquid cathode (FLC-dc-APGD), was utilized before for the production of PAL designated for future agricultural uses.

Thus, we undertook the development of a procedure for the acquisition of PAL of defined physicochemical properties, by implementing the design of experiments (DOE) followed by response surface methodology (RSM) approaches intended for a multivariate optimization of the operating parameters of the FLC-dc-APGD-based reaction-discharge system. The continuous-flow character of the FLC-dc-APGD system developed by our research group may be considered a novelty in contrast of the majority of previous studies on the stationary reaction-discharge systems [45,46]. In addition, the herein applied multivariate optimization approach is innovative in comparison to the one-factor-at-time (OFAT) method frequently used at the optimization stage for evaluating the impact of the discharge gas flow rate [45] or the gas mixture [45] on the physicochemical composition of the analyzed liquids. Here, we established the optimal operating conditions for continuous production of PAL based on the response surface regression models. For the first time, we demonstrated antibacterial properties of PAL against plant pathogens of high economic importance, which, in this case, belong to two diverse SRP species, namely *Dickeya solani* and *Pectobacterium atrosepticum*. The observed antimicrobial action of plasma-treated solution was attributed, according to the detailed qualitative and quantitative analyses, to RONS and solvated electrons (e^−^_aq_) produced in PAL. In summary, this work is a response to a challenge associated with the effective production of PAL of required antibacterial and physicochemical properties, which will be easy to collect and store. A pioneering character of this research results from the usage of the reaction-discharge system working in a flow-through mode for an efficient generation of a fertilizer-based PAL of potent antibacterial properties, which were documented for the first time toward SRP.

## 2. Results and Discussion

### 2.1. Response Surface Regression Models Describing the Effect of the Operating Parameters of the FLC-dc-APGD System on Electrical Conductivity of the Resultant NH_4_NO_3_-Based PAL

All experimental treatments listed in the Box-Behnken Design (BBD) matrix (see Table 1) were carried out in one block, according to the randomized run order. σ_1h_ and σ_24h_ of all the resultant PALs were measured after 1 and 24 h, respectively. Since three independent aliquots were taken for each sample treatment, means of σ_1h_ and σ_24h_ for each PAL were calculated along with variances for these three-point measurement series. Initially, scatter plots of the previously mentioned variances versus the respective means of σ_1h_ and σ_24h_ were used to judge whether variability of the means of σ_1h_ and σ_24h_ between treatments was greater than variability of these means within single treatments. Scatter plots of the means of σ_1h_ and σ_24h_ versus the randomized run order were used to look for any patterns or trends. Since variability of the means of σ_1h_ and σ_24h_ between treatments was higher than variability within these treatments, and no trends or patterns were observed in both datasets, it was concluded that the differences in the measured values of σ_1h_ and σ_24h_ resulted only from changes in the settings of the operating parameters of the FLC-dc-APGD system.

Means of σ_1h_ and σ_24h_, assessed for the Box-Behnken response surface design were fitted next with full quadratic functions, including linear (A, B, C), square (A^2^, B^2^, C^2^), and combined (A × B, A × C, B × C) terms. A backward-elimination-of-terms algorithm at α = 0.05 was used to select only these terms that were statistically significant and contributed to the changes in σ_1h_ and σ_24h_ of PALs produced in the FLC-dc-APGD-based reaction-discharge system. The model hierarchy was kept in this case, leaving all lower-order terms that comprised higher-order terms. Statistically significant terms in the regression models of σ_1h_ and σ_24h_ along with the respective *p*-values confirming this significance and the lack-of-fit test are gathered in Table 2. Detailed analyses of variance (ANOVA) summaries with the statistics for both response surface regression models are given in Table 3. Values of R^2^, adjusted R^2^, and predicted R^2^ are included in Table 2 to point out goodness-of-fit and the forecast performance of both models.

Regression models of σ_1h_ and σ_24h_ were statistically significant since *p*-values equaled 0.000 (see Table 2). *p*-values for the lack-of-fit test were higher than α = 0.05, particularly in case of the model for σ_24h_, confirming that there were no reasons to reject both regression models. R^2^ values, showing the degree to which the selected operating parameters explained variance in the collected datasets, were higher than 99%. All the above-listed measures pointed out that the developed response surface regression models properly described the relationships between the examined parameters of the FLC-dc-APGD system and the σ_1h_ and σ_24h_ measures for PALs produced with this system. High values of the predicted R^2^ indicated that both models showed the ability to reliably predict responses for a new set of the operating parameters. Interestingly, in the case of both models (Table 2), the square terms were insignificant, while the term B, i.e., the discharge current of the FLC-dc-APGD system, did not contribute to the overall values of σ_1h_ and σ_24h_.

**Figure 1:**
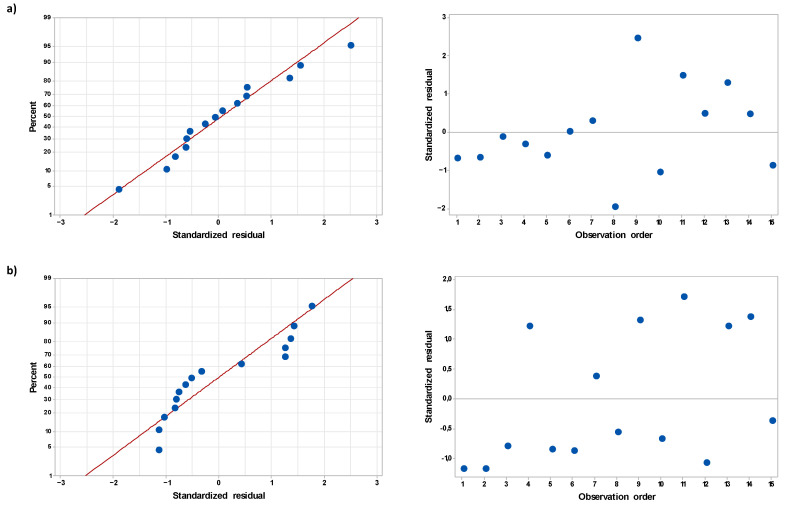
Normal probability plots and scatter plots of residuals versus observation (run) order for surface regression models predicting electrical conductivity of PALs produced using the continuous-flow FLC-dc-APGD system, as measured after (**a**) 1 h (σ_1h_) and (**b**) 24 h (σ_24h_).

Finally, residual plots, i.e., normal probability plots and scatter plots of regular residuals versus the run order for both regression models confirmed their goodness-of-fit (Figure 1). Fairly normal distribution of the residuals in the case of the normal probability plots in addition to random and uncorrelated patterns of the residuals in the scatter plots versus the run order additionally indicated that the examined operating parameters of the FLC-dc-APGD system used for the production of PALs affected the measured σ_1h_ and σ_24h_ in a systematic way.

### 2.2. Selection of the Optimal Experimental Conditions for Production of the NH_4_NO_3_-Based PALs of the Highest Electrical Conductivity

The ANOVA statistics of the developed response surface regression models of σ_1h_ and σ_24h_, as measured in PALs produced in the continuous-flow FLC-dc-APGD reaction-discharge system, fitted the measured data well and described variation in both responses due to changes in the operating parameters of the applied reaction-discharge system. For that reason, both regression models were used for selecting a combination of the settings of the operating parameters of the FLC-dc-APGD system that was enabled to produce PALs of the highest σ_1h_ and σ_24h_. For this purpose, desirability functions of σ_1h_ and σ_24h_, i.e., d(σ_1h_) and d(σ_24h_), were applied and the values of these functions at given settings of the operating parameters of the reaction-discharge system provided the composite desirability (D) value. The latter D value was used to point how well a certain combination of the parameters satisfied the optimization goal, i.e., production of PALs with maximal σ_1h_ and σ_24h_ values. The highest values of d(σ_1h_), d(σ_24h_), and D, being 0.992, 0.957, and 0.974, respectively, were found for the following combination of the experimental parameters: A = 2.0 mL min^−1^, B = 30 mA, and C = 0.50%. For these optimal operating parameters, the values of σ_1h_ and σ_24h_ of PALs were predicted by the models to be 8.347 ± 0.111 mS cm^−1^ and 8.535 ± 0.130 mS cm^−1^, respectively. Both values were within the range of the lower and the upper value of σ measured in the experiment, i.e., 2.10–8.40 mS cm^−1^ for σ_1h_ and 2.12–8.82 mS cm^−1^ for σ_24h_.

**Figure 2:**
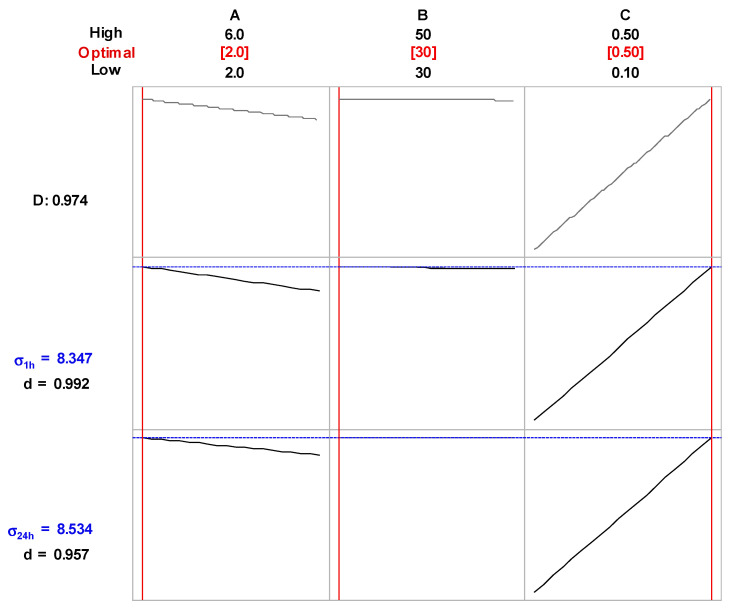
Optimization plots–the effect of the flow rate of the FLC solution (**A**), the discharge current of the FLC-dc-APGD system (**B**), and the NH_4_NO_3_ concentration (**C**) on electrical conductivity of PALs produced in the continuous-flow FLC-dc-APGD system, as measured after 1 h (σ_1h_) and 24 h (σ_24h_).

The optimization plot, showing how different settings of the operating parameters affect the predicted response of σ_1h_ and σ_24h_, are given in Figure 2. It appears that an increase of the flow rate of the FLC solution (parameter A) in both models resulted in a gradual decrease of the σ_1h_ and σ_24h_ values, hence, in a lower production rate of the long-lived reactive nitrogen species (RNS) like: NO_2_^−^, NO_3_^−^, and NH_4_^+^. This could be likely the consequence of a relatively shorter time of exposure of the surface of the FLC solution to the discharge when the flow rate of the FLC solution increased. Similar results concerning the effect of the flow rate of the FLC solution on the concentration of the generated NO_2_^−^, NO_3_^−^, and NH_4_^+^ ions in a comparable discharge system were previously reported by Jamroz et al. [47]. In the latter device, in spite of an increase in the energy yield of the discharge, the water evaporation rate was reduced and, hence, the concentration of water vapor and H_2_O^+^ ions in the discharge phase was also diminished. As a result, the concentration of the products of dissociate electron-recombination of the H_2_O^+^ ions and electron-impact dissociation of the water molecules themselves, i.e., •H and •OH radicals as well as other O reactive species had to be lower in these conditions as well [48,49]. The main source of NO_2_^−^, NO_3_^−^, and NH_4_^+^ ions in the liquid treated by APGD systems operated in contact with this solution playing the role of the cathode of the discharge system, are nitric oxide (NO), nitric dioxide (NO_2_), and nitric hydride (NH) species, respectively [47,48]. These di-atomic and tri-atomic molecules readily react with •OH and •H radicals in the discharge phase according to the following reactions: NO + OH = HNO_2_, NO_2_ + OH = HNO_3_, NH_x_ + H = NH_x+1_, where x = 0–2 [48,50]. When the concentration of •H and •OH radicals in the discharge phase is decreased, the concentration of the NO, NO_2_, and NH molecules is also lower because they are obtained through reactions between active N_2_ molecules with the above-mentioned •H radicals and free active O radicals [47,48,50]. Surprisingly, the effect of the discharge current (parameter B) was negligible. Certainly, water evaporation from the surface of the FLC solution as well as acceleration of different particles in the discharge phase is likely higher when the discharge current is increasing [51]. In these conditions, collision processes are more intensive and the population of the excited species rises, as measured by different optical temperatures [51]. Finally, augmentation in the concentration of NH_4_NO_3_ in the FLC solution led to a linear increase in σ, likely as a result of the elevation in the concentration of electric charge carriers in the liquid.

The appropriateness of the optimal operating parameters of the FLC-dc-APGD system for the production of NH_4_NO_3_-based PALs with the highest values of σ_1h_ and σ_24h_ was verified in the additional independent experiment. Accordingly, solutions of NH_4_NO_3_ were treated by FLC-dc-APGD in the applied continuous-flow reaction-discharge system, operated under the above-defined conditions, i.e., A = 2.0 mL min^−1^, B = 30 mA, and C = 0.50%. The electrical conductivity of the collected aliquots of the resultant PALs measured after 1 and 24 h equaled 8.94 mS cm^−1^ for σ_1h_ and 9.11 mS cm^−1^ for σ_24h_, respectively. These values turned out to be comparable to those predicted by the developed regression models. Relative errors between them were 7% (σ_1h_) and 6% (σ_24h_). Therefore, the reliability of both response surface regression models and the appropriateness of the optimal operating conditions of the FLC-dc-APGD system for the production of the NH_4_NO_3_-based PALs of pre-defined σ have been confirmed.

Since there are several operating parameters, including the discharge current, the flow rate, and the concentration of the utilized precursor, which might have an effect on the physicochemical properties of the resultant PAL, a multivariate optimization involving DOE along with RSM was applied to find the optimal operating conditions for the production of the PAL of certain desirable properties. In comparison to previous works [45,46], this kind of the multiparameter optimization approach allowed us not only to limit the number of the required experiments, but also to estimate prospective interactions between the studied operating conditions and their effect on physicochemical properties of the PAL. In such a way, efficient production of the PAL having the required physicochemical parameters (e.g., conductivity) could have been achieved.

### 2.3. Antibacterial Action Against Plant Pathogens of 0.5% NH_4_NO_3_ Treated in the Optimized FLC-dc-APGD System

It is worth noticing that both species selected for this study, namely *P. atrosepticum* and *D. solani*, belong to the highly homogenous [52,53,54,55] representatives of the *Pectobacteriaceae* family. *P. atrosepticum* strains have been commonly isolated from seed potato fields in temperate climate regions for more than a century [56], while *D. solani* is believed to be an emerging pathogen of high economic importance [7].

The 24 h exposure of *D. solani* IFB0099 and *P. atrosepticum* IFB5103 cells to 25% or 50% PAL was potent enough to inhibit the growth of the investigated phytopathogens in TSB (Trypticase Soy Broth) medium (Table 4). The application of 1% and 10% PAL did not result in growth inhibition of the tested plant pathogenic bacteria (Table 4). The included control samples did not only prove a lack of the antibacterial properties of the 0.5% NH_4_NO_3_ solution not treated by plasma, but also confirmed the viability of the utilized bacterial cells in addition to the sterility of the solutions, the water, and the media used. Therefore, *D. solani* IFB0099 and *P. atrosepticum* IFB5103 turned out to be highly susceptible to the plasma-treated 0.5% NH_4_NO_3_ solution with minimal inhibitory concentration (MIC) equaling 25% (Table 4).

The incubation (24 h, 28 °C) of *D. solani* IFB0099 or *P. atrosepticum* IFB5103 suspensions on TSA (Trypticase Soy Agar) media after subjection of bacterial cells either to 25% or 50% PAL for 24 h resulted in the observed absence of bacterial colonies (Table 4). However, the utilization of 1% or 10% PAL in an analogous experiment was not effective enough to kill the cells of the investigated phytopathogens (Table 4). The utilized control samples did not only demonstrate a lack of bactericidal action of the 0.5% NH_4_NO_3_ solution untreated by plasma, but also proved the viability of bacterial cells in addition to the sterility of the solutions, the water, and the media used. Thus, 25% concentrated PAL was potent enough to eradicate the studied phytopathogens and was designated as minimal bactericidal concentration (MBC) (Table 4). In other words, 100% (1−2 × 10^6^) cells of *D. solani* IFB0099 and *P. atrosepticum* IFB5103 included in this experiment lost viability due to 24 h of exposure to the developed PAL.

Among phytopathogens previously subjected to the action of PALs, the causative agents of fungal diseases dominate [35,37,38]. Wu et al. [35] utilized either oxygen or air as working gases to obtain PAWs in a corona plasma jet-based system. The resultant efficacy of *Colletotrichum gloeosporioides* inactivation reached 56% or 96% (post 10 or 30 min of the NTAP treatment, respectively) in terms of the air-PAW, while the action of the oxygen-PAW resulted in a loss of cell viability in 15% or 55% (after 10 or 30 min of exposure, respectively). Regarding the work of Siddique et al. [37], a Dyne-A-Mite HP surface treatment plasma machine was utilized for the generation of PAWs, based on tap, deionized, or distilled water, that were subjected to the action of the discharge either for 30 or 60 min. From the obtained PAWs, the ones based on deionized and distilled water post 60 min exposure to plasma that were diluted to 50% concentration turned out to successfully inhibit germination of the phytopathogenic conidia [37]. Moreover, Guo et al. [38] applied a dielectric barrier discharge (DBD) plasma device for the acquisition of PAW15, PAW30, PAW45, and PAW60, named according to the provided irradiation time. Statistically significant, i.e., 0.75, 1.3, and 3.3 log reductions resulted from 30 min of incubation in PAW30, PAW45, and PAW60 of *Penicillium italicum* conidia that had been attached to the surface of kumquats. No notable variations in the color, thickness, or the total contents of ascorbic acid, flavonoids, and carotenoids in the fruit have been noted [38]. To the best of our knowledge, from the group of bacterial plant pathogens, solely *Xanthomonas vesicatoria*, has been subjected to the action of PAL before [36]. In that study, no direct antibacterial action of water activated by DBD was noted in vitro. However, the enhancement of host plant defense systems leading to the limitation of the disease symptoms development on tomatoes was observed [36]. In view of the former research, we demonstrated, for the first time, antibacterial properties of PAL toward economically important bacterial phytopathogens. The higher susceptibility to PAL of soft rot *Pectobacteriaceae* in contrast to *Xanthomonas vesicatoria* might not only be associated with the plasma source and experimental setup, but also the utilized microbial density and culture state in addition to such apparent factors as the studied species and even the strain [57]. It was previously established that antibacterial properties of PALs have been associated with changes in pH, oxidation-reductive potential, UV radiation, shock waves, photons, electric fields, and, most of all, the generated RONS [17,28,58]. Since extensive characterization and elucidation of the complex plasma chemistry is required not only to reveal the antimicrobial mechanism of action of the obtained PAL, but also to make a step in reaching a generally regarded as safe (GRAS) status [17,26] for the developed formulation, a profound physicochemical study of the produced RONS has been conducted.

### 2.4. Examination of Interactions and Processes Leading to Acquisition of NH_4_NO_3_-Based PAL of the Defined Physicochemical and Antibacterial Properties

The following qualitative and quantitative measurements of RONS, present in the FLC-dc-APGD-treated 0.5% NH_4_NO_3_ solution in contrast to the unexposed control sample, have been performed.

**Figure 3:**
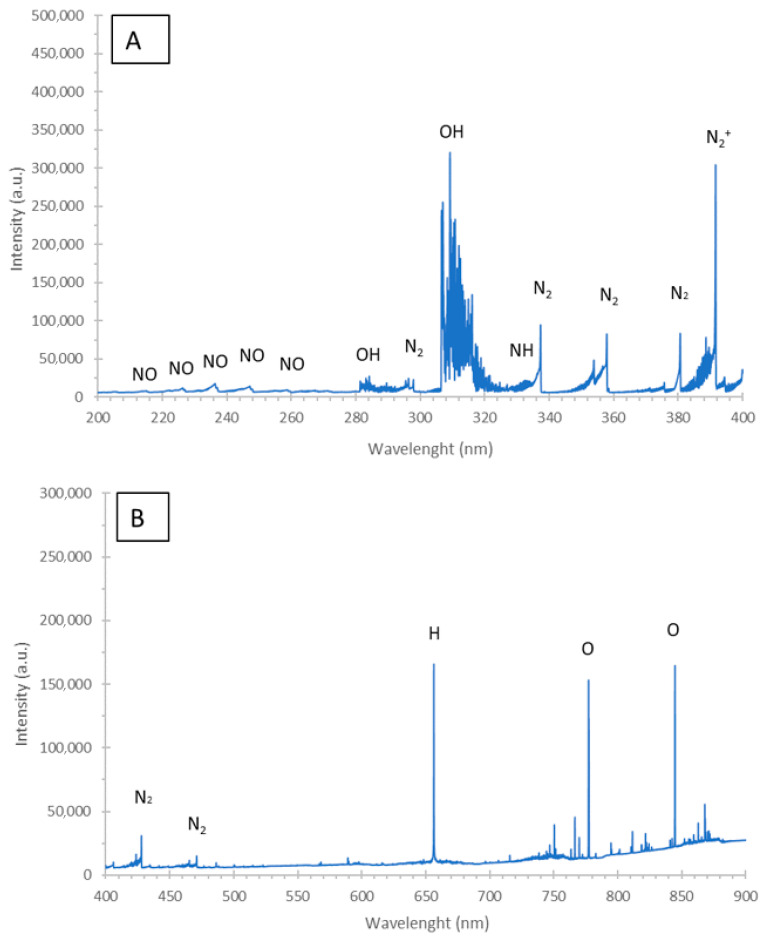
The optical emission spectra acquired in two spectral ranges: (**A**) 200–400 nm and (**B**) 400–900 nm during the FLC-dc-APGD treatment of the 0.5% NH_4_NO_3_ solution under the optimal operating conditions.

In qualitative evaluation, optical emission spectrometry (OES) (Figure 3) was applied to identify numerous bands of NO and N_2_ molecules, belonging to the γ-system (A^2Σ+^-X^2^Π) and the second positive system (C^3^Π_u_-B^3^Π_g_), respectively. Additionally, two strong bands of OH molecules (with heads at 281.1 nm (1-0) and 306.4 nm (0-0), belonging to the A^2^Σ-X^2^Π system), as well as the band of NH molecules (with the head at 336.0 nm, belonging to the A^3^Π_g_-X^3^Σ^−^ (0-0) system), were detected. The band of N_2_^+^ of the first negative system (B^2^Σ^+^_u_-X^2^Σ^+^_g_) (with heads at 391.4 nm (0-0) and 427.1 nm (0-1)) was also observed. In the ranges of 400–500 nm and 550–900 nm, several bands of N_2_ molecules, belonging to the second positive system (C^3^Π_u_-B^3^Π_g_) and first positive system (B^3^Π_g_–A^3^Σ^+^_u_), respectively, were clearly visible. Moreover, two lines of hydrogen (H), i.e., the strong one Hα at 656.28 nm and a very weak Hβ at 486.13 nm, in addition to O lines (at 777.2, 772.4, and 844.6 nm) were identified. Based on these OES results, it has been concluded that the following RONS: OH, NH, N_2_, NO_x_, N_2_^+^, and O, were excited in the plasma (gas) phase of FLC-dc-APGD.

Subsequently, quantitative analyses of the selected RNS were carried out for 0.5% NH_4_NO_3_ treated solution under optimal operating conditions by FLC-dc-APGD, in contrast to the untreated control sample. In the case of all investigated RNS, including NO_2_^−^, NO_3_^−^, and NH_4_^+^, their concentrations were shown to change after the exposure of the solution to FLC-dc-APGD. The content of NO_2_^−^ increased twice (from 4.5 to 9.3 mg L^−1^) post the NTAP action. In addition, the concentration of NO_3_^−^ was elevated from 1195 to 1624 mg L^−1^ after the FLC-dc-APGD-treatment. Similarly, the concentration of NH_4_^+^ was augmented from 710 to 957 mg L^−1^ due to the applied treatment.

**Figure 4:**
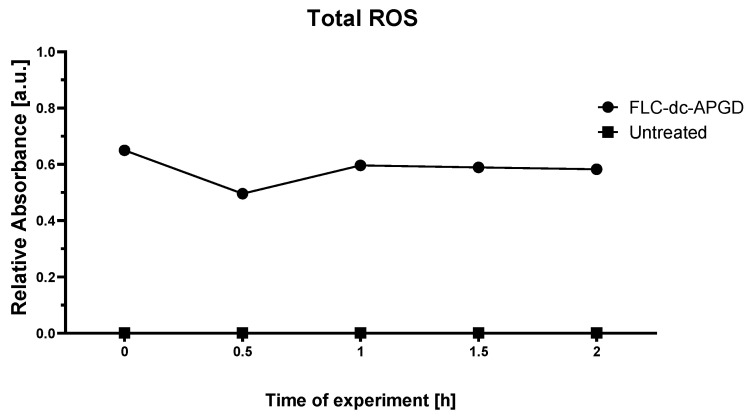
The absorbance measured at 590 nm for the PAL produced in the FLC-dc-APGD-based reaction-discharge system and the untreated 0.5% NH_4_NO_3_ solution.

Then the total amount of reactive oxygen species (ROS, Figure 4), involving OH, O, O_3_, H_2_O_2_, and HO_2_, was determined in the NTAP-treated 0.5% NH_4_NO_3_ solution with the use of the color-forming reaction with the KI-starch reagent [59]. It was found that the absorbance of the PAL-KI starch system remained stable over time after the NTAP operation (a mean absorbance value was ≈ 0.6), suggesting that the long-lived ROS were present in this solution at the concentration of 28.79; 19.76; 25.63; 25.21; 24.85 mg L^−1^ at subsequent time points. As expected, the measured absorbance for the control sample (untreated 0.5% NH_4_NO_3_ solution) was close to 0 and unchanged in time (Figure 4).

**Figure 5:**
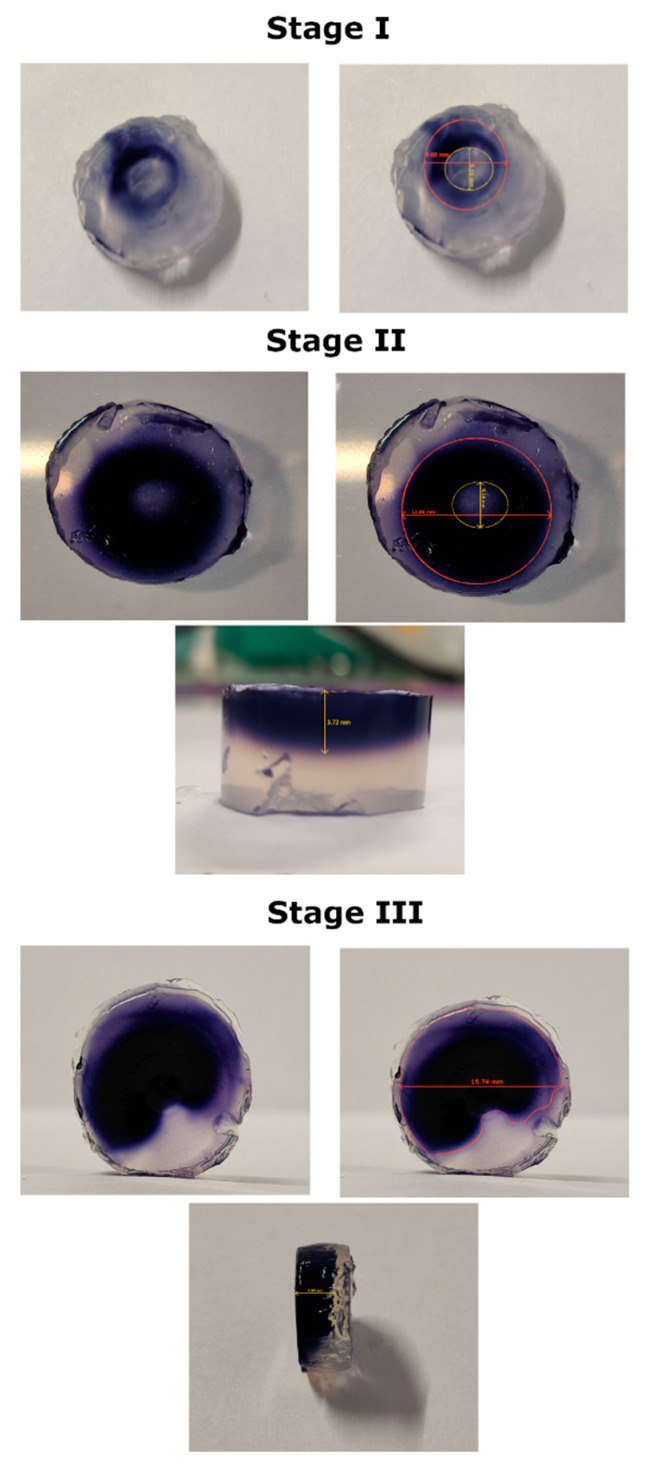
Spatial distribution of ROS in the KI-starch gels. Stage I—photograph from initial generation of FLC-dc-APGD (1 s). Stage II—changes recorded after an average time (2 s) of FLC-dc-APGD generation in a flowing mode. Stage III—variation resulting from a prolonged (4 s) FLC-dc-APGD irradiation. Prior to NTAP exposure, the gels had been covered with a droplet of 0.5% (*m*/*v*) NH_4_NO_3_. Navy blue tint results from effective ROS generation. The gels were photographed from the front, back, and side views.

To delve further into the topic of ROS generated in the resulting PAL and establish the spatial distribution of these molecules in the volume of the liquid (Figure 5), the interactions between FLC-dc-APGD and a droplet of the 0.5% NH_4_NO_3_ solution placed on the KI-starch gel were studied. At the beginning of the action of FLC-dc-APGD (stage I, Figure 5) initiated under optimal operating conditions (the flow rate of solution = 2.0 mL min^−1^, the discharge current = 30 mA, and NH_4_NO_3_ concentration = 0.50% (*m*/*v*)), the oxidation process occurred in a ring-shaped pattern characterized by the intact and colored areas of 5.14 mm and 9.60 mm in diameter, respectively. The penetration depth equaled in this case ~1 mm. Therefore, the oxidation processes occurred in the first layers. The expansion of the previously mentioned ring-shaped region was observed following the prolongation of the contact between the 0.5% NH_4_NO_3_ solution placed on KI-starch gel and FLC-dc-APGD for the average time in the flowing regime (stage II, Figure 5). In this case, the diameter of the central intact area did not change. However, the colored region enlarged to 11.88 mm. Furthermore, the penetration depth through the gels was higher, reaching 3.72 mm. Finally, the most prominent spatial distribution of the oxidative area was detected after the prolonged NTAP treatment (stage III, Figure 5), leading to generation of a colored area of 15.74 mm without any intact fragment. The produced ROS penetrated the KI-starch gel deeply (5.63 mm). In essence, the NTAP exposure led to the generation of ROS in the entire volume of the irradiated droplets, leading to the acquisition of a stable in time PAL of high oxidative potential. To the best of our knowledge, spatial distribution of total ROS generated during the NTAP operation in the continuous-flow reaction-discharge system has not been examined yet. However, the resultant ring-shaped oxidative area typically occurs in other NTAP sources, e.g., in a DBD system [60] or in an atmospheric pressure plasma jet [61], presenting similar patterns and a comparable penetration depth into the KI-starch gel.

A synergistic antibacterial action of the detected ROS and RNS, i.e., OH, O, O_3_, H_2_O_2_, HO_2_, NH, N_2_, N_2_^+^, NO_2_^−^, NO_3_^−^, and NH_4_^+^ might be postulated. ROS destroy microbial cell membranes, cell wall, DNA, RNA, proteins, carbohydrates, and other components of the intracellular machinery [17,28,62]. Active and structural biomolecules are oxidized and chemical bonds within peptidoglycan of the cell wall break. Cytoplasmic leakage resulting from the loss of integrity of the depolarized outer membrane follows. Regarding RNS, these radicals trigger not only lipid peroxidation associated with the loss of a hydrogen atom from a methylene group, leading to cross-linkage of the fatty acid side chain and creation of pores in the cellular membrane [62], but also oxidize tyrosine residues of proteins, thiols, and unsaturated fatty acids [17]. It is worth considering that NTAP-generated RONS might reach the inner part of the bacterial cell by active transport as well as due to infiltration through pores resulting from the lipid peroxidation process. An intracellular concentration of the active species may also rise post subjection to oxidative and nitrosative stresses [19,62].

Notably, the varying antibacterial impact of the PAL-related RONS in the case of human pathogens was attributed to Gram classification of the treated microbe, physiological condition of the culture, and whether planktonic cells or biofilms were exposed to the plasma-activated media [28]. Different bacterial responses to active species may also be linked with diverse efficacy of microbial oxidative and nitrosative stress response systems [19], which rely on specific enzymes, including catalases, peroxidases, and superoxide dismutases, in addition to the production of smaller molecules like thioredoxin, glutaredoxin, or glutathione [28]. Importantly, the herein reported RONS of a long-lived character were demonstrated to penetrate deeply into the activated liquid and the included *D. solani* and *P. atrosepticum* strains were unable to tackle this threat.

Referring to future applications, a high-voltage NTAP source, i.e., FLC-dc-APGD, operated in the continuous-flow reaction-discharge system has been utilized for the generation of PAL of defined physicochemical properties. In more detail, the type and concentration of the acquired RONS may be controlled by the current-voltage parameters. Due to the operation of the reaction-discharge system under the stated optimal conditions (discharge current: 50 mA, concentration of NH_4_NO_3_: 0.5% (*m*/*v*), flow rate of solution: 2.0 mL min^−1^), a high-throughput production of PAL (120 mL h^−1^) has been achieved. It is worth underlining that 1 mL of PAL was subjected to FLC-dc-APGD just for 30 s, which was a much shorter exposure time in comparison to other devices used for the production of the previously reported PALs of antimicrobial properties [37,38]. Furthermore, reaching physicochemical stability of the developed PAL was necessary to aim for the application of the developed active solution not only for decontamination, but also for the extension of storage time and shelf life of agricultural goods and other food products [58].

## 3. Materials and Methods

### 3.1. Reagents and Solutions

As a PAL solution precursor, ammonium nitrate NH_4_NO_3_ (Archem, Kamieniec Wroclawski, Poland) was used. To perform quantitative analyses of RONS, produced under the optimal operating conditions of PAL fabrication, commercially available reagents, i.e., Nitrite HR Reagent HI93708-0 (Hanna Instruments, Salaj, Romania) and Nitrate Reagent HI93728-0 (Hanna Instruments, Salaj, Romania) along with the Nessler’s reagent (Pol-Aura, Zabrze, Poland), potassium iodide (Avantor Performance Materials, Gliwice, Poland), starch (Sigma-Aldrich, Steinheim, Germany), and agar (BTL, Lodz, Poland) were used. Re-distilled water was used throughout. All of the reagents were of an analytical grade or better.

### 3.2. Production of the Plasma-Treated Liquid by FLC-dc-APGD

**Figure 6:**
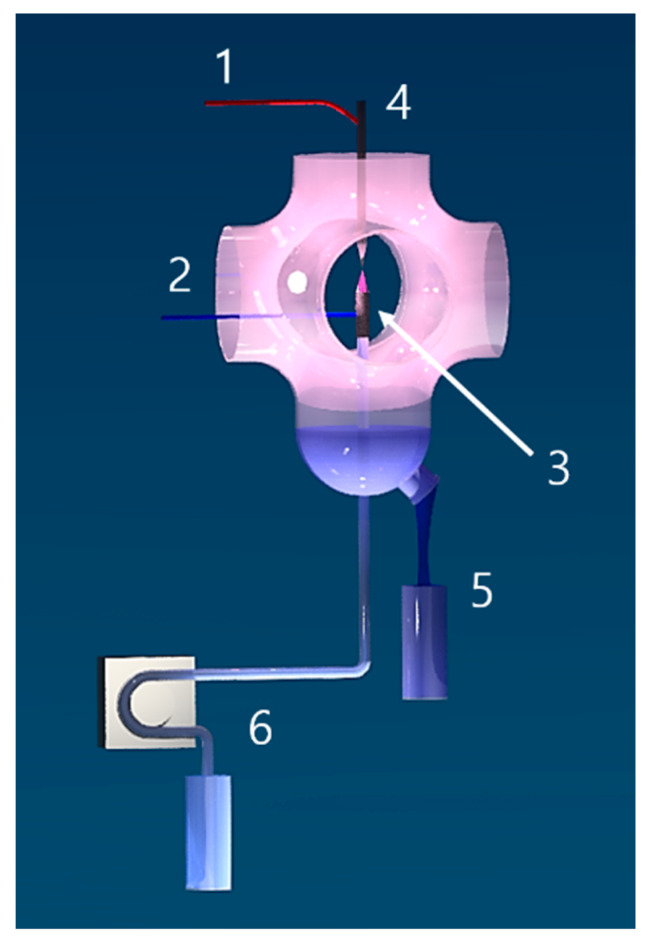
The NTAP-based reaction-discharge system used for PAL production. A layout of the NTAP-based reaction-discharge system applied for continuous production of PAL. (1)—negative potential supplied to a metallic anode. (2)—positive potential supplied to a flowing liquid cathode through a Pt wire. (3)—FLC-dc-APGD being a NTAP source. (4)—a metallic anode. (5)—PAL. (6)—a four channel peristaltic pump used for pumping the NH_4_NO_3_ solution.

The PAL was obtained in the continuous flow reaction-discharge system, previously developed by our group [63], employing FLC-dc-APGD as an NTAP source (Figure 6). In more detail, FLC-dc-APGD was generated in a 2.0 mm gap between a pin-type tungsten anode and NH_4_NO_3_ solution of a defined concentration (0.1–0.5% (*m*/*v*)) acting as the FLC. The flow rate of the introduced solution depended on the working conditions of the system, as shown in detail in the BBD matrix (Table 1). To control the flow rate of the NH_4_NO_3_ solution, a four-channel peristaltic pump (Masterflex L/S, Cole Palmer, Vernon Hills, IL, USA) was used. To link this pump with a discharge chamber, a silicone hose was connected to a quartz capillary (OD = 4.00 mm) onto which a graphite tube (OD = 6.0 mm) was mounted. Additionally, a platinum wire was attached to the quartz-graphite tube and connected to HV dc power supply (Dora Electronics Equipment, Wroclaw, Poland), which enabled us to set a certain discharge current value (30–50 mA, according to the BBD matrix, see Table 1). The discharge current values of 30–50 mA were stabilized by applying a 10 kΩ ballast resistor (Tyco Electronics, USA). The supplied voltage was maintained in the range of 1100–1400 V. After exposure of the NH_4_NO_3_ solution to the FLC-dc-APGD operation, 10 mL of such NTAP-treated NH_4_NO_3_ solution was collected to polypropylene tubes (Sarstedt, Numbrecht, Germany) and stored at 4 °C for further analyses.

### 3.3. Multiparameter Optimization of the Operating Parameters of FLC-dc-APGD System

In order to produce PAL of defined electrical conductivity (*σ*, in mS cm^−1^) in the proposed continuous-flow reaction-discharge system, the operating parameters of this system were optimized by a multiparameter approach, i.e., the RSM, using a Minitab 17 software. A BBD was used for this purpose and the optimization procedure looked as follows: (a) all experiments according to treatments listed in the BBD matrix (Table 1) were carried out, (b) response surface regression models expressing changes of the response, i.e., *σ* of the resulting PALs measured after 1 and after 24 h, were developed, (c) abilities to describe the measured values of σ_1h_ and σ_24h_ and predict new values by these models were assessed, and, finally, (d) the optimal settings of the operating parameters of the reaction-discharge system providing the highest values of σ_1h_ and σ_24h_ for the produced PALs were selected.

The operating parameters, initially verified to have an effect on physicochemical properties of the PAL and selected for the optimization experiment by the BBD, were as follows: the flow rate of the NH_4_NO_3_ solution (A, in mL min^−1^), the discharge current of FLC-dc-APGD sustained in contact with the NH_4_NO_3_ solution (B, in mA), and the concentration of NH_4_NO_3_ in this solution (C, in %). The response surface design included 15 randomized treatments (see Table 1) at three different levels (codded values in brackets) of the previously mentioned parameters, i.e., 2.0 mL min^−1^ (−1), 4.0 mL min^−1^ (0), and 6.0 mL min^−1^ (+1) for A, 30 mA (−1), 40 mA (0), and 50 mA (+1) for B, and 0.1% (−1), 0.3% (0), and 0.5% (+1) for C. Three center points were included in this response surface design. Ranges of the operating parameters were selected to provide a stable and reproducible operation of the FLC-dc-APGD system. All treatments were carried out in one block. For each treatment, three portions (10 mL) of the PAL produced at the given experimental conditions were collected into polypropylene vials (Sarstedt, Numbrecht, Germany) and, then, average values of σ_1h_ and σ_24h_, measured in PALs with the use of an Elmetron CPC-505 pH/conductivity-meter (Zabrze, Poland), were used to develop appropriate response surface regression models. σ_1h_ and σ_24h_ were fitted with full quadratic functions with linear (A, B, C) and square (A^2^, B^2^, C^2^) terms of the studied parameters, and their two-way interaction terms (AB, AC, BC). A general equation for the response surface regression models were given as: σ = c_0_ + c_1_ × A + c_2_ × B + c_3_ × C + c_11_ × A^2^ + c_22_ × B^2^ + c_33_ × C^2^ + c_12_ × A × B + c_13_ × A × C + c_23_ × B × C, where c_0_ is the intercept while c_1_–c_33_ are regression coefficients. A backward-elimination-of-terms algorithm at α = 0.05 was used to select statistically significant terms in these equations describing σ_1h_ and σ_24h_.

Reliability of the developed response surface regression models for both responses, i.e., σ_1h_ and σ_24h_, was checked by using ANOVA. In this case, goodness-of-fit (a degree of explanation of variance in measured responses by input variables) of established modeling functions was assessed through coefficients of determination (R^2^). Their general explanatory power was evaluated by adjusted R^2^ values, while the prediction power (ability to predict responses for new sets of variables) was given by the predicted R^2^ values. In addition, *p*-values were given to point out the statistical significance (at α = 0.05) of the regression models, and certain linear, square, and two-way interaction terms included in them. Finally, residuals associated with the developed regression models were visually analyzed to find any clear heteroscedasticities and non-normalities (normal probability plots) or correlations and trends (scatter plots of normalized residuals versus the run order).

The optimal settings of the operating parameters, leading to continuous production of PALs with the highest values of σ_1h_ and σ_24h_ in the applied flow-through reaction-discharge system, were selected on the basis of values of the individual d(σ_1h_) and d(σ_24h_) functions. The values of these functions changed from 0 (no satisfaction of the optimization goal) to 1 (full satisfaction of the optimization goal). The optimization goal was established in order to find such a set of parameters at which both σ_1h_ and σ_24h_ would be maximized. Hence, the D, being [d(σ_1h_) × d(σ_24h_)]½, was used to find this set of parameters and operate the FLC-dc-APGD system to produce NH_4_NO_3_-based PALs of pre-defined physicochemical properties in a controlled manner.

### 3.4. Assessment of Antibacterial Properties of 0.5% NH_4_NO_3_ Activated in the Optimized FLC-dc-APGD System

Plant pathogenic bacteria (Table 5) utilized for evaluating antimicrobial properties of the 0.5% NH_4_NO_3_-based PAL were kept as frozen stocks in 40% glycerol at −80 °C at the Intercollegiate Faculty of Biotechnology University of Gdansk and Medical University of Gdansk in Poland.

Prior to the experiment, the inoculation loop full of the bacterial biomass (Table 5), collected from the frozen stock, was streaked on a Trypticase Soy Agar (TSA, BTL, Poland). This culture was conducted for 24 h at 28 °C. Subsequently, a single bacterial colony was picked from the TSA plate and utilized for inoculation of 5 mL of a Trypticase Soy Broth (TSB, BTL, Poland) liquid medium. TSB containing bacterial cells was incubated at 28 °C with 120 rpm shaking for 24 h in order to obtain the overnight bacterial culture of a given strain. This culture was then centrifuged for 10 min at 6500 rpm and the resultant bacterial pellet was washed twice in sterile 0.85% NaCl solution. Afterward, the optical density of bacterial suspension was adjusted to 0.5 in McFarland (McF, approx. 1−2 × 10^8^ colony forming units mL^−1^ [66]) scale with a densitometer DEN-1B (Biosan, Latvia).

MIC defined as the concentration of the active substance potent enough to prevent multiplication of microorganisms, in addition to MBC, referring to the formulation that causes loss of bacterial cell viability due to the action of 0.5% NH_4_NO_3_ solution treated in the optimized operating conditions of the FLC-dc-APGD system, were determined in 96 well-plates as follows. A total of 90 µL of the TSB medium, 10 µL of the 0.5 McF suspension of the tested phytopathogenic cells (Table 5), prepared as given above, and 100 µL of the NTAP-treated 0.5% NH_4_NO_3_ solution dissolved in sterile distilled water to reach the final concentration of 1%, 10%, 25%, or 50%, were introduced to each well. Post preparation, basal absorbance of the contents of the wells was measured with an EnVision 2105 Multimode Plate Reader (PerkinElmer, Waltham, MA, USA). The 96-well plates were incubated for 24 h at 28 °C and the absorbance measurement was then repeated. The lowest concentration of NTAP-treated 0.5% NH_4_NO_3_ allowing for inhibition of the bacterial growth was stated as MIC. Afterward, 5 µL of the contents of the wells that showed no visible growth of the studied microorganism was plated on TSA medium. If bacterial colonies did not grow after a 24 h incubation at 28 °C, the lowest concentration allowing for such an observation was described as MBC. This experiment was conducted in three repetitions with each consisting of two technical repeats. Concerning control samples, MIC and MBC assays have also been performed without the addition of PAL (either non-treated by plasma 0.5% NH_4_NO_3_ solution or water utilized for diluting PAL was applied to the 96-wells plate instead). Moreover, controls evaluating the viability of bacterial cells (TSB medium + 0.5 McF bacterial suspension) or the sterility of the utilized components (just TSB medium, TSB + PAL, TSB + 0.5% NH_4_NO_3_ solution, TSB + water used for dilutions, TSB + 0.85% NaCl utilized for preparation of bacterial suspensions) were included in the MIC and MBC procedures.

### 3.5. Plasma Reactive Species Responsible for the Antibacterial Action of the PAL

To decipher the mechanism of action of the NTAP-treated 0.5% NH_4_NO_3_ against plant pathogenic bacteria, the following qualitative and quantitative analyses were performed.

The RONS, which have been generated in the NH_4_NO_3_ solution treated by FLC-dc-APGD under the optimal operating conditions, were qualitatively identified by OES. The OES spectrum was acquired in the range from 200–900 nm with a Shamrock SR-500i (Andor, Belfast, United Kingdom) instrument, supported by a Newton DU-920P-OE CCD camera (Andor, Belfast, United Kingdom). The radiation emitted by FLC-dc-APGD during the production of the plasma-treated 0.5% NH_4_NO_3_ solution was collimated onto a 10 µm slit. The gaining time of the CCD camera was set to 0.50 s. The Solis software (version 2.5) was used for data acquisition and analysis.

In the subsequent quantitative analyses of RNS, the concentrations of NO_2_^−^ and NO_3_^−^ generated in the NTAP-treated 0.5% NH_4_NO_3_ solution, in contrast to the untreated control, were assessed immediately after exposure of the liquid to dc-APGD. Here, manufacturer’s protocols attached to the commercial colorimetric assays, respectively, HANNA HI 96,708 and HANNA HI 96,728 (Hanna Instruments, Salaj, Romania), were used. The Nessler’s method [47] was applied to determine the concentrations of NH_4_^+^ in the PAL produced under the optimal conditions and the corresponding untreated control sample. The absorbance of the product of a reaction between ammonia and the Nessler’s reagent, i.e., [(Hg-O-Hg)NH_2_], was measured at 420 nm by using an Analytik Jena Specord 210 Plus UV/Vis absorption spectrophotometer (Jena, Germany).

Moreover, the method described by Uchida et al. [59] was utilized for the estimation of the total concentration of ROS. In more detail, 2.9 mL of the tested 0.5% NH_4_NO_3_ solution (either NTAP-activated under the optimal operating conditions or the untreated control sample) was mixed with 100 µL of a KI-starch reagent solution, consisting of a 0.3% potassium iodide solution and 0.5% starch solution [59] post (t1 = 0 h; t2 = 0.5 h; t3 = 1 h; t4 = 1.5 h; t5 = 2 h) NTAP-treatment of the experimental sample. In these reactions, the KI-starch suspension acts as the source of I^−^ ions, reacting with all ROS present in the analyzed samples, whose oxidative potential is higher than this for iodide (0.54 V). The presence of starch in the reactant suspension results in a deeper blue tint of the starch-iodide complex exposed to ROS. The total amount of oxidative agents generated during NTAP exposure was evaluated from absorbance values at 600 nm with the use of Analytik Jena Specord 210 Plus UV/Vis absorption spectrophotometer (Jena, Germany). According to the measured absorbance values at 600, the quantitative analysis of the total ROS concentration was based on to a five-point calibration curve. For this purpose, the standard H_2_O_2_ solution was serially diluted, obtaining: 1.75, 4.3, 8.25, 12.75, and 17 mg L^−1^ H_2_O_2_ working solutions. Next, the prepared solutions were mixed with the KI-starch reagent in the same manner as was previously described and the absorbance values at 600 nm were acquired.

Finally, the spatial distribution of total ROS concentration was visualized with the use of a KI-starch gel as reported by Uchida et al. [59]. For this purpose, a KI-starch reaction suspension was prepared by dissolution of 0.3% (*m*/*v*) potassium iodide, 0.5% (*m*/*v*) starch, and 1.2% agar (*m*/*v*) (BTL, Lodz, Poland) [59] in the volume of 200 mL of deionized water. Subsequently, the mixture was heated under ambient air conditions without reaching the boiling point. Afterward, the prepared KI-starch reaction suspension was poured on standard Petri dish plates to obtain 8 mm thick solid disks from which 19 mm-diameter smaller disks have been cut. The resultant single disk has been placed in the central part beneath the FLC electrode (Figure 6, (3)-FLC-dc-APGD) and its surface has been wetted with the droplet of non-treated 0.5% (*m*/*v*) NH_4_NO_3_ solution to ignite NTAP. The impact of FLC-dc-APGD on ROS generation was captured as colorimetric changes in three stages: (1) at the moment of glow discharge ignition (1 s), (2) post an average contact (2 s) with droplets of NH_4_NO_3_ solution in a flowing mode, and (3) after prolonged (4 s) NTAP action. An increase in total ROS concentration was recognized by the occurrence of a dark blue tint appearing on the KI-starch disks.

## 4. Conclusions

In view of a high need for novel, innovative, and eco-friendly plant protection methods against SRP, a multivariate optimization of FLC-dc-APGD-based reaction-discharge system allowed us to define the optimal operating parameters, i.e., flow rate of the FLC as 2.0 mL min^−1^, the discharge current as 30 mA, and the inorganic salt concentration in the FLC solution as 0.50, to be utilized for a high-throughput production of NH_4_NO_3_-based PAL of the highest conductivity. The developed PAL exhibited antibacterial properties toward *D. solani* and *P. atrosepticum* basing on the established MIC and MBC values of 25%. We attributed the antimicrobial action of PAL to the following RONS: OH, O, O_3_, H_2_O_2_, HO_2_, NH, N_2_, N_2_^+^, NO_2_^−^, NO_3_^−^, and NH_4_^+^. Since the generated PAL is easy to store and transport, it may find numerous applications in the agricultural sector, such as limiting the occurrence and spread of devastating bacterial phytopathogens.

## 5. Patents

Patent no. 236665 attributed on 13.10.2020 by the Patent Office of the Republic of Poland resulted from the work reported in this manuscript.

## Figures and Tables

**Table 1:** Box-Behnken response surface design with actual and (coded) values of operating parameters related to application of a continuous-flow FLC-dc-APGD reaction discharge system for the production of NH_4_NO_3_-based PALs having certain electrical conductivity measured after 1 (*σ*_1h_) and 24 h (*σ*_24h_).

Order		A, mL min^−1^	B, mA	C, %	σ _1h_, mS cm^−1^	σ _24h_, mS cm^−1^
Standard	Run					
11	1	6.0 (+1)	40 (0)	0.1 (−1)	2.230	2.310
5	2	2.0 (−1)	30 (−1)	0.3 (0)	5.180	5.230
13	3 ^a^	4.0 (0)	40 (0)	0.3 (0)	5.000	5.200
7	4	6.0 (+1)	30 (−1)	0.3 (0)	4.810	5.060
4	5	4.0 (0)	50 (+1)	0.5 (+1)	7.960	8.090
2	6	4.0 (0)	50 (+1)	0.1 (−1)	2.240	2.260
15	7 ^a^	4.0 (0)	40 (0)	0.3 (0)	5.130	5.270
3	8	4.0 (0)	30 (−1)	0.5 (+1)	7.800	7.880
10	9	2.0 (−1)	40 (0)	0.5 (+1)	8.400	8.820
6	10	2.0 (−1)	50 (+1)	0.3 (0)	5.120	5.170
8	11	6.0 (+1)	50 (+1)	0.3 (0)	5.320	5.340
12	12	6.0 (+1)	40 (0)	0.5 (+1)	7.510	7.880
9	13	2.0 (−1)	40 (0)	0.1 (−1)	2.230	2.270
14	14 ^a^	4.0 (0)	40 (0)	0.3 (0)	5.240	5.300
1	15	4.0 (0)	30 (−1)	0.1 (−1)	2.100	2.120

A: The flow rate of the FLC solution (NH_4_NO_3_) (in mL min^−1^). B: The discharge current of the FLC-dc-APGD system (in mA). C: The NH_4_NO_3_ concentration in the FLC solution (in %). ^a^ Center points at A = 4.0 mL min^−1^ (0), B = 40 mA (0), C = 0.3% (0).

**Table 2:** *p*-values for response surface regression models as well as linear and square effects of parameters A, B, and C along with their two-way interactions included in these models to describe changes in σ_1h_ and σ_24h_ (in mS cm^−1^) of PALs produced by using the continuous-flow FLC-dc-APGD reaction-discharge system. Statistically significant terms included in the developed regression models are given in brackets.

	*p*-Values					R^2^, %	R^2^ Adjusted, %	R^2^ Predicted, %	S
	Model	Linear	Square	Two-Way Interactions	Lack-of-Fit				
*σ_1h_*	0.000	0.000 (A, C)	-	0.016 (A × C)	0.070	99.5	99.4	98.7	0.173
*σ_24h_*	0.000	0.000 (A, B, C)	-	0.004 (A × B, A × C)	0.626	99.8	99.7	99.3	0.114
		Regression equations modelling the effect of examined parameters (A, B, and C) ^a^							
*σ_1h_*		0.258−0.128 × A + 17.269 × C−0.613 × A × C							
*σ_24h_*		1.159−0.184 × A−0.019 × B + 16.519 × C + 0.007 × A × B + 0.556 × A × C							

σ_1h_—Electrical conductivity measured after 1 h. σ_24h_—Electrical conductivity measured after 24 h. A: The flow rate of the FLC solution (NH_4_NO_3_) (in mL min^−1^). B: The discharge current of the FLC-dc-APGD system (in mA). C: The NH_4_NO_3_ concentration in the FLC solution (in %). R^2^: Coefficient of determination. S: Residual standard deviation. ^a^ A backward-elimination-of-terms algorithm at α = 0.05 was applied to determine statistically significant terms in response to surface regression models.

**Table 3:** ANOVA statistics and results of the lack-of-fit test for the response surface regression models established using the backward-elimination-of-terms algorithm (at α = 0.05) for production of PALs in the continuous-flow FLC-dc-APGD reaction-discharge system.

Source of Data	DF	Adjusted SS	Adjusted MS	F-Value ^a^	*p*-Value
Electrical conductivity measured after 1 h (σ_1h_)					
Model	3	70.612	23.537	785.36	0.000 < 0.05
Linear	2	65.172	32.586	1087.29	0.000 < 0.05
A	1	0.101	0.101	3.28	0.093
C	1	65.071	65.071	2171.20	0.000 < 0.05
Two-way interactions	1	0.240	0.240	8.01	0.016 < 0.05
A×C	1	0.240	0.240	8.01	0.016 < 0.05
Error	11	0.330	0.030		
Lack-of-fit	9	0.324	0.036	13.69	0.070 > 0.05
Pure error	2	0.005	0.003		
Total	14	70.942			
Electrical conductivity measured after 24 h (σ_24h_)					
Model	5	65.870	13.174	1006.94	0.000 < 0.05
Linear	3	60.683	20.228	1546.08	0.000 < 0.05
A	1	0.221	0.221	16.92	0.003 < 0.05
B	1	0.024	0.024	1.84	0.208
C	1	60.399	60.399	4616.53	0.000 < 0.05
Two-way interactions	2	0.279	0.140	10.67	0.004 < 0.05
A×B	1	0.081	0.081	6.21	0.034 < 0.05
A×C	1	0.198	0.198	15.14	0.004 < 0.05
Error	9	0.118	0.013		
Lack-of-fit	7	0.089	0.013	0.88	0.625 > 0.05
Pure error	2	0.029	0.014		
Total	14	65.987			

DF: Degrees of freedom. SS: The sum of squares. MS: The mean of squares. A: The flow rate of the FLC solution (NH_4_NO_3_). B: The discharge current of the FLC-dc-APGD system. C: The NH_4_NO_3_ concentration in the FLC solution. ^a^ The value of the F-test for comparing model variance with residual variance.

**Table 4:** Antibacterial properties of the plasma-treated 0.5% NH_4_NO_3_ solution against phytopathogens.

Bacterial Strain	Assay	Concentration of PAL			
		1%	10%	25%	50%
*Dickeya solani* IFB0099	MIC	+	+/−	-	-
	MBC	+	+/−	-	-
*Pectobacterium atrosepticum* IFB5103	MIC	+	+/−	-	-
	MBC	+	+/−	-	-

PAL: Plasma-activated liquid. MIC: Minimal inhibitory concentration. MBC: Minimal bactericidal concentration. +: growth of bacterial cells was observed/lack of antibacterial action. −: no bacterial growth/antibacterial properties were observed. The experiment was repeated three times with two technical repetitions in each. The following control samples were included: MIC and MBC assays performed without the addition of PAL (either 0.5% NH_4_NO_3_ solution or water utilized for diluting PAL was applied instead). In addition, control samples evaluating the viability of bacterial cells (TSB medium + 0.5 McF bacterial suspension) or sterility of the used components (just TSB medium, TSB + PAL, TSB + 0.5% NH_4_NO_3_ solution, TSB + water applied for dilutions, TSB + 0.85% NaCl used for the preparation of bacterial suspensions) were utilized in the MIC and MBC procedures.

**Table 5:** Phytopathogenic bacteria used in this study.

Bacterial Species	Strain Nos ^a^	Disease Caused	Host	Year of Isolation	Country of Isolation	Reference
*Dickeya solani*	IFB0099, IPO2276, LMG28824.	Blackleg and soft rot	*Solanum tuberosum*	2005	Poland	Slawiak et al. [64]
*Pectobacterium atrosepticum*	IFB5103, SCRI1086.			1985	Canada	SCRI collection [65]

^a^ The listed numbers originate from the following international bacterial collections: IFB—collection of Intercollegiate Faculty of Biotechnology University of Gdansk and Medical University of Gdansk (Gdansk, Poland), IPO—collection of the Institute for Phytopathological Research (Wageningen, The Netherlands), LMG—collection of the Laboratory of Microbiology in Gent (Gent, Belgium), SCRI—The James Hutton Institute bacterial collection (Dundee, Scotland).

## Abbreviations

ANOVA	analysis of variance
BBD	Box-Behnken Design
D	composite desirability
DBD	dielectric-barrier discharge
dc-APGD	direct current atmospheric pressure glow discharge
DOE	design of experiments
FLC	flowing liquid cathode
GRAS	generally regarded as safe
MBC	minimal bactericidal concentration
McF	McFarland
MIC	minimal inhibitory concentration
NTAP	non-thermal atmospheric pressure plasma
OES	optical emission spectrometry
OFAT	one-factor-at-time
PAL	plasma-activated liquid
PAW	plasma-activated water
RONS	reactive oxygen and nitrogen species
ROS	reactive oxygen species
RNS	reactive nitrogen species
RSM	response surface methodology
SRP	soft rot *Pectobacteriaceae*
TSA	Trypticase Soy Agar
TSB	Trypticase Soy Broth
